# Novel Etching Technique for Delineation of Prior-Austenite Grain Boundaries in Low, Medium and High Carbon Steels

**DOI:** 10.3390/ma13153296

**Published:** 2020-07-24

**Authors:** Richard Thackray, Eric J. Palmiere, Omar Khalid

**Affiliations:** Department of Materials Science and Engineering, The University of Sheffield, Portobello Street, Sheffield S1 3JD, UK; e.j.palmiere@sheffield.ac.uk (E.J.P.); okhalid@mac.com (O.K.)

**Keywords:** steel, etching, grain boundaries, austenite, microstructure

## Abstract

The etching of prior austenite grain boundaries in martensite for detailed quantitative metallography of low to high carbon steel has been carried out using aqueous solutions of picric acid containing different wetting agents. The choice of wetting agent was shown to be dependent on the carbon content of the steel, with sodium dodecyl sulfate (SDS) being more suitable for use with low and medium carbon steels, whereas sodium dodecylbenzene sulfonate (SDBS) was shown to be more appropriate for high carbon steels. It is also recommended that, for a particular steel, a variety of temper treatments should be carried out in order to reveal grain boundaries, particularly where more detailed results than simple grain size measurements are required. Finally, the use of dummy specimens prior to etching of the real samples was shown to reduce the need for re-polishing and re-etching of the samples.

## 1. Introduction

Etching is basically a controlled corrosion process resulting from the electrolytic action between surfaces of different potential [1]. One of the more difficult structures to etch is that of prior-austenite grain boundaries in as-quenched steels, such that the martensitic structure is represented as a low contrast background against a higher contrast set of grain boundaries. The revealing of prior-austenite grain boundaries in steels is important because the end properties of steels are influenced by the grain structure developed during the reheating and/or thermomechanical processing which occurs in the austenite. Several different methods and techniques such as chemical etching and thermal etching exist to reveal the prior austenite grain boundaries [1,2,3,4,5,6,7]. These methods will be described below, together with the associated advantages or problems. 

### 1.1. Chemical Etching 

There are many influencing factors when it comes to the etching of prior austenite grain boundaries. These include the type of wetting agent used, the hydrochloric acid concentration, the solution concentration, the temperature and the swabbing technique. 

Saturated aqueous picric acid solution is most commonly used to reveal prior-austenite grains because it produces a slow, uniform dissolution of the ferrite lamella with the cathodic cementite in relief but suppresses ferrite grain boundaries. Nonetheless, it is difficult to reveal the prior-austenite grain boundaries because no one single etchant is used for all samples. To obtain a successful etch there should be a chemical attack on the boundaries but not on the matrix. Tempering the specimen can aid in improving etch response without affecting the grain size; as it is a well-known phenomenon that impurities (e.g., P, Sn, Sb and As) segregate to prior austenite grain boundaries [8,9] when held for long periods from 1 to 100 h, in the temperature range 350–600 °C [10,11]. The study of segregation of impurity elements to grain boundaries has been studied using Auger electron spectroscopy to investigate the cause of temper embrittlement. Barraclough [2], in 1973, reviewed the use of different etching solutions to outline the prior austenite grain boundaries in 0.42 wt.% carbon steel with a martensitic microstructure obtained by quenching. Specimens were etched in the quenched state and after tempering at different temperatures for different durations. Barraclough concluded that to produce a consistently sufficent delineation of the prior-austenite grain boundaries, the specimen should be tempered for two hours at a temperature of 625 °C, then furnace cooled to 550 °C and held for 72 h before finally being water quenched. He also concluded that the picric acid solution works best when heated to 85 °C. A similar method was employed by Mahajan, et al. [12] in which the samples were etched in boiling water saturated with picric acid. In both cases the solutions were based on saturated aqueous picric acid plus a wetting agent (which is used to change the surface energy of a liquid or solid surface and influence interfacial interactions) [13]. The wetting agent generally utilised is sodium alkyl sulphonate “Teepol”(sodium alkyl sulphonate is known as Teepol in the United Kingdom). Nelson, [14] evaluated the effect of five wetting agents for the delineation of the prior-austenite grain boundaries using saturated aqueous picric acid, and found four different responses by evaluating the effect of increasing the concentration of the wetting agents: (i) a maximum limit is observed where a noticeable sharp improvement occurs; however, over this limit higher concentrations of the wetting agent do not produce an effect. (ii) There is a slight gradual improvement with increasing the concentration of the wetting agent. However above the maximum limit higher concentrations are detrimental. (iii) The wetting agents have little or no value. (iv) The wetting agent is counterproductive in any concentration or damaging with increased concentration. 

### 1.2. Oxidation Etching 

This technique reveals the prior-austenite grain boundaries by relying on the fact that oxidation accumulation preferentially occurs along the grain boundaries or by grain boundary decarburization (reduction of carbon) [1]. This method requires the steel specimen to be slightly polished prior to placing it in an electrically heated furnace with an oxidizing atmosphere. So that oxidation can occur on the required side, the polished side should be facing upwards. Once the sample has been held at the required temperature for the desired hold time, the sample is water quenched to form a martensitic structure. Careful removal of the oxide layer must be performed during the grinding and polishing stage, as excessive grinding could remove the affected layers of oxide. The drawback of this method is that at high austenitization temperatures, the bulk diffusion rate is too high to preferentially oxidize the grain boundaries. However, at temperatures below 1038 °C [1], the grain boundary diffusion permits selective oxidation as it predominates.

### 1.3. Thermal Etching

The thermal etching method consists of heating a steel sample which has been finely pre-polished with a 1 µm diamond paste, in an inert atmosphere or a vacuum. A vacuum pressure of at least 1 Pa or higher is needed to avoid oxidation [3,4]. The sample is heated to the austenitization temperature and then cooled down to room temperature. The prior austenite grain boundaries are shown by grooves which decorate them, these grooves at the grain boundaries are formed by matter transport and surface tension effects. The rate at which the specimens are cooled from the austenitization temperature has an effect on the grooves which decorate the prior austenite boundaries. When the cooling rate is too slow, ghost traces of the grooves occur. This in turn leads to differences between the inner and outer grain size, therefore leading to false measurements of the prior austenite grain size. Furthermore, during slow cooling from temperatures higher than 1200 °C, the austenite grain can continue its growth, therefore complicating the accuracy of the prior austenite grain boundary measurements [4]. 

### 1.4. Other Methods

A direct method used to observe the prior austenite grain boundaries at high temperatures is a high temperature microscope [5]. Another method used by many researchers to reveal the prior austenite grain boundaries is the precipitation of ferrite, cementite and fine pearlite [1,6]. Vander Voort (1984) [1] has reported that, when successfully employed, it is a reliable method for austenite grain size measurements; the drawback of this is that considerable experimentation is generally required before reliable results can be obtained. 

As described above, delineation of the prior austenite grains can be achieved by many techniques. In this paper, a chemical etching technique for high purity carbon steels, using two new wetting agents has been developed for the etching of prior austenite grain boundaries.

## 2. Materials and Methods 

The investigation was carried out on 12 silicon-killed steel samples with three different carbon chemistries. Low (0.08 wt.% C) shown in Table 1, medium (0.38 wt.% C) shown in Table 2, and high (0.8 wt.% C) shown in Table 3. Compositions were measured by inductively coupled plasma (ICP) and optical emission spectroscopy (OES) techniques. Within each of these three groups, the niobium concentration varied from 0 to 0.02 wt.%. 

**Figure 1:**
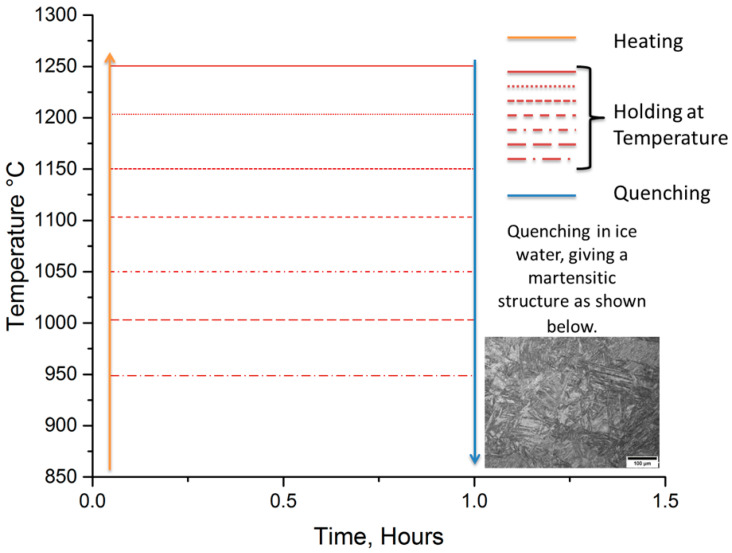
Schematic of the reheating temperature and quenching process used for the three carbon steels with varying niobium concentration to examine the microstructural evolution. Holding time was one hour, and heat treatments were carried out in an argon atmosphere.

After the heat treatment shown in Figure 1 all the specimens were mounted, ground and polished to 1 µm ready to be etched. During the etching procedure, a pH meter (Omega PHH-37, Egham, Surrey, UK) was placed in the solution to obtain the pH value when the first 3 dummy specimens were each placed in the solution for 5 min. The successful etching solution was produced by dissolving picric acid into 100 mL of distilled water which was heated and maintained at 80 °C. It is important to keep stirring the picric acid until the distilled water is completely saturated with picric. The second step is to add a few drops of hydrochloric (HCl) acid into the solution and then to add the wetting agents. HCl and the three wetting agents, sodium dodecyl sulfate, sodium dodecylbenzene and sodium alkyl sulphonate were used in the etching of all of the samples discussed in this study. A total of 4 drops of HCl and 1 g of wetting agent were used throughout. Trials were carried out using as little as 0.5 g and as much as 5 g of the wetting agent, but the best results were obtained for 1 g. As mentioned above, an important point to note is that the solution was matured by using 3 dummy specimens which were etched for 5 min each (15 min in total). 

### Atomic Force Microscopy 

Atomic force microscopy was conducted on the etched specimens using a Dimension 3100 AFM (Bruker, Billerica, MA, USA), using a tapping technique to obtain the image. To obtain a high-resolution image, the scan rate was set at 0.5 Hz and the tip velocity was 30 µm/s. 

## 3. Results

### 3.1. Sodium Alkylate Sulfonate (Teepol)

**Figure 2:**
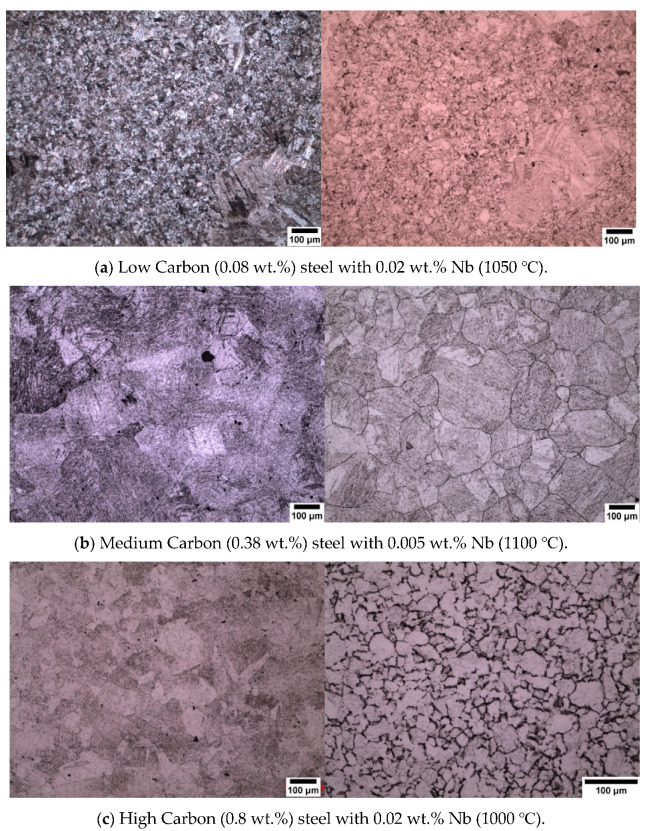
Steel specimens etched with saturated picric acid with different wetting agents. Sodium alkylate sulfonate (Teepol) in the left-hand column, (**a**–**c**), Sodium dodecyl sulfate (SDS) in the right-hand column (**a**,**b**), and Sodium dodecylbenzene sulfonate (SDBS) in the right-hand column (**c**).

All three different carbon contents are shown in Figure 2. All the specimens were quenched in iced water from the temperatures shown below the micrographs. All the starting microstructures were initially martensitic, and it can be seen that it is difficult to distinguish between the prior austenite grain boundaries and the inner martensitic structure. It can be seen from these micrographs that the addition of sodium alkylate sulfonate (Teepol) gives poor results which do not outline the prior austenite grain boundaries, therefore, making it extremely difficult to measure the prior austenite grain size of the specimen. 

### 3.2. New Wetting Agents

**Figure 3:**
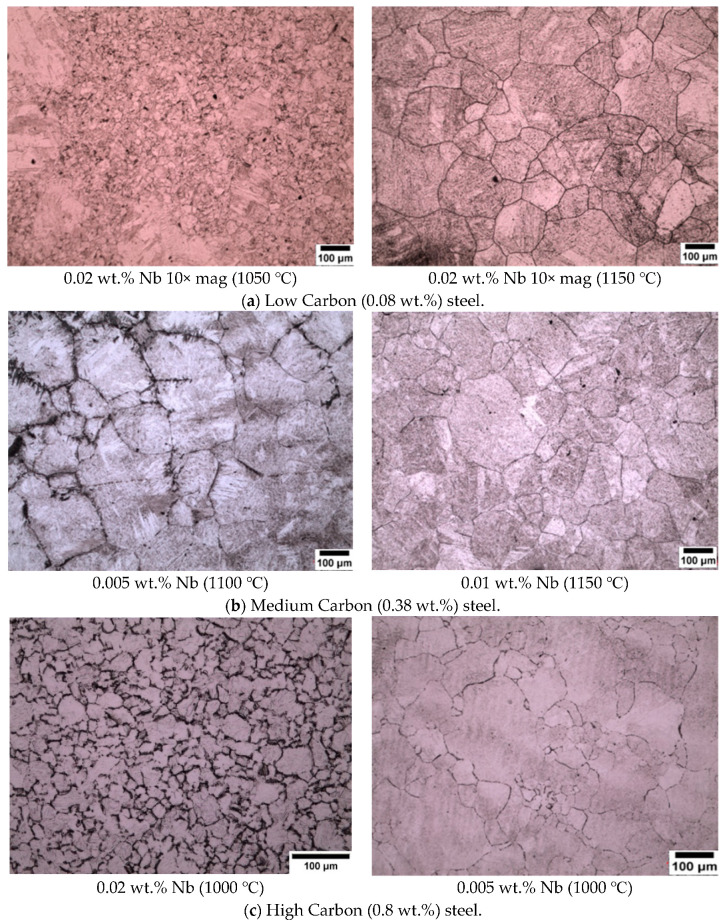
Steel specimens etched with saturated picric acid with sodium dodecyl sulfate (SDS) as a wetting agent used for (**a**,**b**) and sodium dodecylbenzene sulfonate for (**c**).

As reported in Section 3.1, conventional wetting agents performed poorly when attempting to outline prior austenite grains, hence other wetting agents were trialled. The two new wetting agents used in this work are sodium dodecyl sulfate (SDS) and sodium dodecylbenzene sulfonate (SDBS). The results of using these agents can be seen in the micrographs presented in Figure 3. An important point to note is that sodium dodecyl sulfate (SDS) is an effective wetting agent for low carbon (0.08 wt.%) and medium carbon (0.38 wt.%) steel specimens but did not reveal any prior austenite grain boundaries in the high carbon (0.8 wt.%) steel specimens. SDBS was the most effective wetting agent for the high carbon (0.8 wt.%) steel specimens, as can be seen in Figure 3c. 

**Figure 4:**
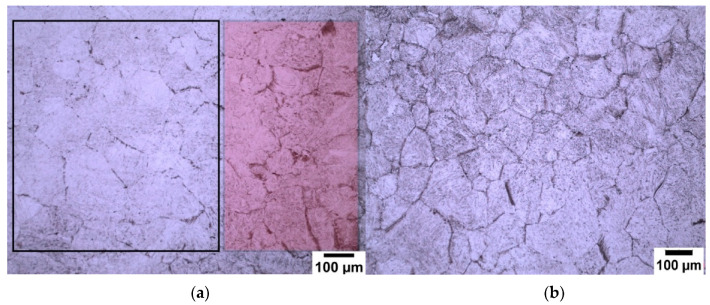
Etching layer effect on 0.8 wt.% carbon steel with 0.005 wt.% Nb at 1100 °C. (**a**) shows the effect of etching layer thickness on the definition of PAGBs and (**b**) shows how the delineation of PAGBs should be when the etching layer is evenly distributed.

It is important to note that when the specimen is submerged in the saturated picric solution it should be not be moved as this affects the etching layer that forms on the specimen surface causing one side to be thicker and the other side to be thinner. In Figure 4a the black outline represents the area where the etching layer was thin, and the red shaded area is where the etching layer is thicker as the grain boundaries are much more clearly defined. Figure 4b shows the light optical micrograph of how the delineation of the prior austenite grain boundaries should be when the etching layer is evenly distributed on the surface. 

**Figure 5:**
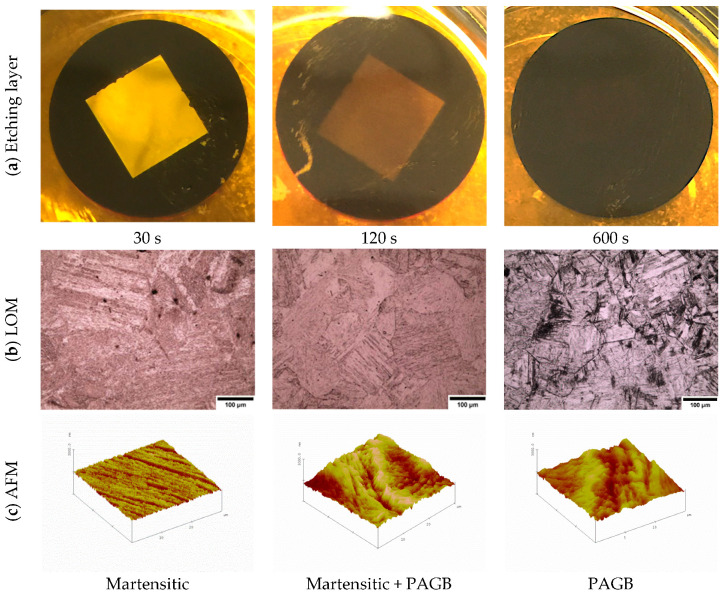
The effect of submerging the specimen for different time durations in saturated picric acid with sodium dodecyl sulfate added. Samples shown here are 0.8 wt.% carbon with 0.005wt.% niobium. (**a**) shows the etching layer on the specimen surface getting thicker with increased time, (**b**) shows the effect of time on the etched microstructure and (**c**) shows the topography of the specimen surface at each time duration.

It is important to note that for every time interval a fresh solution was made, and the same specimen was ground and polished again to keep the experimental work accurate as possible. Figure 5 illustrates the effect of the etchant layer on the specimen with increasing time, submerged under saturated picric acid. It can be seen in the first row, (etching layer) that the etching layer gets thicker with increasing time. The second row (LOM) shows the light optical micrographs of the microstructure obtained when the submersion time was increased. As shown in the LOM micrographs for 30 s submersion time, the martensitic structure is revealed but no prior austenite grain boundaries can be seen. After the sample has been submerged for 120 s the austenite grain boundaries start to be outlined; however, the image is still not clear enough to give reliable results. The best results were obtained by submerging the specimen in the solution for 600 s. In this case, a thick black etchant layer is formed on the steel surface; when this layer has been removed, clear prior austenite grain boundaries can be observed. The third row in Figure 5 shows the topography of the specimen surface at each time interval as characterized by atomic force microscopy (AFM). The results from the atomic force microscopy are presented in more detail in the next section; however, in Figure 5, the topography image for 30 s submersion can be seen to give a rough structure where no indication of prior austenite grain boundaries can be found. A thin groove of the prior austenite grain boundaries can be seen at 120 s in the AFM image of the topography (the dark orange line in the middle). However, the best results are obtained at 600 s showing a deep grooved prior austenite grain boundary which is consistent with the dark prior austenite grain boundaries shown by the light micrograph images. 

The results shown above give a clear indication that the formation of a dark thick etchant layer on the surface of the specimen is beneficial and can be part of the etching mechanism.

### 3.3. Atomic Force Microscopy (AFM)

Atomic force microscopy is used in this study to obtain the topography of the surface of the steel specimen, etched using different wetting agents and for different times which in turn produces a thicker etchant layer. This is to understand in greater detail, the mechanisms of the etching process, especially the time taken to form an etchant layer and the effect of the thickness of the layer formed.

**Figure 6:**
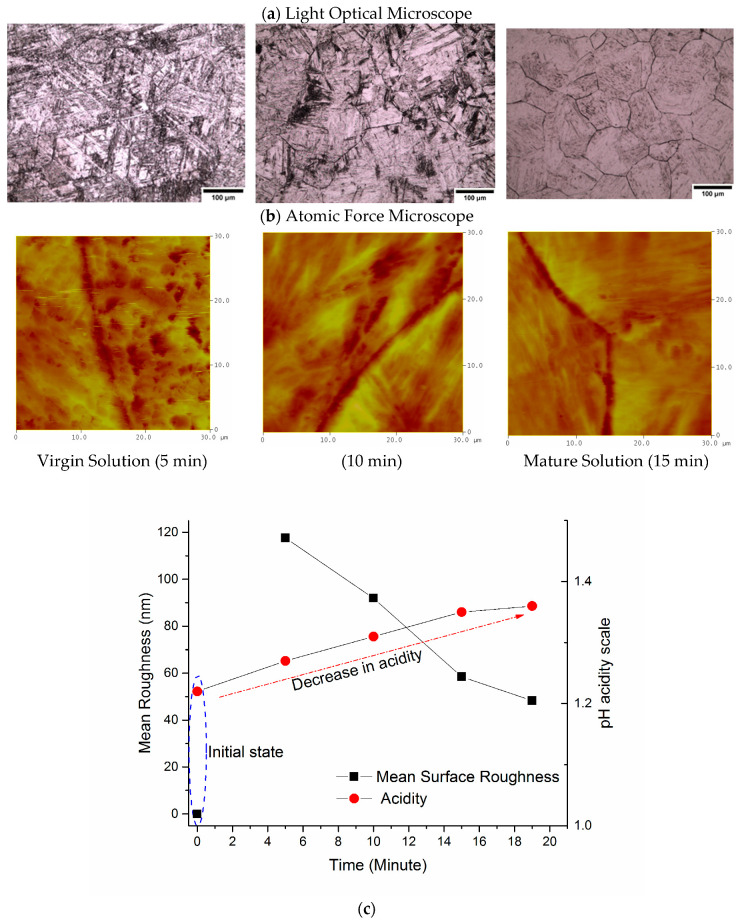
Specimen surface roughness and pH change versus the total time that the etchant solution has been in use. (**a**,**b**) show optical and AFM micrographs of the specimen surface as a function of the total time the etchant has been in use. The graph in (**c**) shows mean surface roughness and pH as a function of the time the etchant has been in use.

Figure 6 shows the light optical and atomic force micrographs of the surface roughness for the specimen etched as a function of the total time the etchant solution has been in use. The graph illustrates data using a pH meter (Omega PHH-37) to measure the acidity of the etchant solution. The pH results indicate that the acidity of the etchant solution decreases with the time that the specimens are being etched in the solution. The initial pH value of the etchant solution starts at 1.22 pH and this increases to 1.27 pH after the first specimen is submerged into the solution for 5 min. As the second specimen is submerged for an additional 5 min the pH of the solution further increases to 1.31 pH. Overall, the pH of the solution increases to 1.36 for a total etching time of 19 min. 

An interesting point to note is that the roughness of the specimen surface after being etched also decreases. This can be seen by comparing the light optical micrographs and the atomic force micrographs in Figure 6. The first specimen etched for 5 min has a mean surface roughness of 117.61 nm indicating that the etchant had attacked the martensitic inner structure and the prior austenite grain boundaries. This is confirmed by the light optical microscope images showing the inner microstructure of the prior austenite grains being etched. As a fresh identical specimen is held for an additional 5 min in the same solution, the mean surface roughness decreases to 91.94 nm giving better results. However, the best results were obtained when the solution was in use for a total of 15 min (matured solution); in this case the mean surface roughness was 58.45 nm and a clear distinction can be made between the inner structure and the prior austenite grain boundaries. 

**Figure 7:**
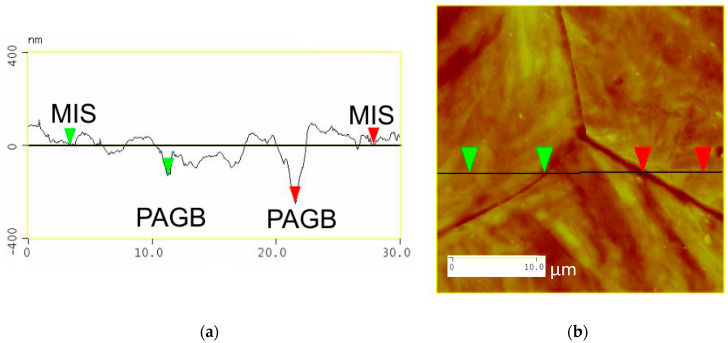
Surface analysis shown in (**a**) and the two-dimensional image shown in (**b**).

Figure 7a illustrates a surface profile analysis for a 30 μm length of the steel surface post etching, showing the martensitic inner structure (MIS) and the prior austenite grain boundaries (PAGB). It should be noted that the PAGB grooves are etched deeper than that of the MIS. Figure 7b shows the image of a triple junction point where three prior austenite grain boundaries meet, this image is obtained from the atomic force microscopy and corresponds to (a) clearly showing the MIS region and the PAGBs which are darker. 

**Figure 8:**
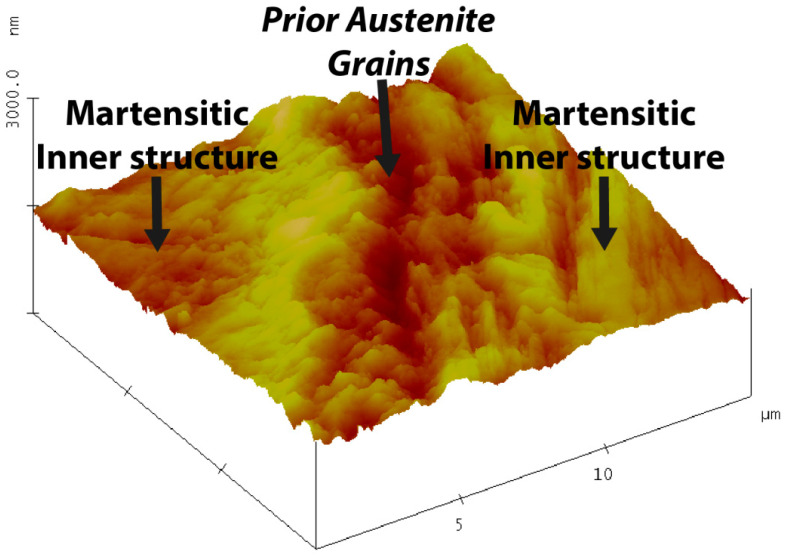
Etched surface image obtained by atomic force microscopy, showing the martensitic inner structure and the prior austenite grain boundaries.

Figure 8 illustrates a 3-dimensional image at a higher magnification analyzing an area of 15 ${µm}^{2}$ of the steel surface post etching. From this figure, the prior austenite grain boundary groove can be seen in the centre and the martensitic inner structure on either side. 

**Figure 9:**
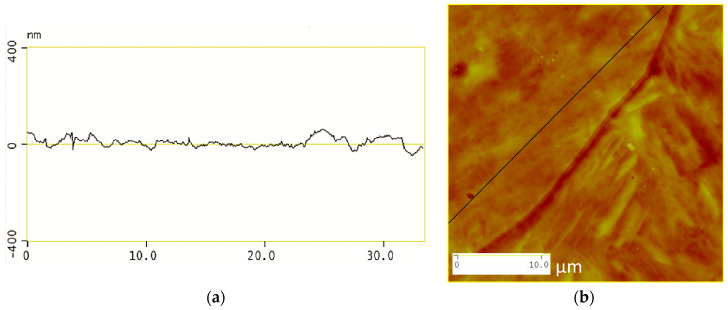
Surface analysis of the martensitic inner structure shown in (**a**) and the two-dimensional image shown in (**b**).

**Figure 10:**
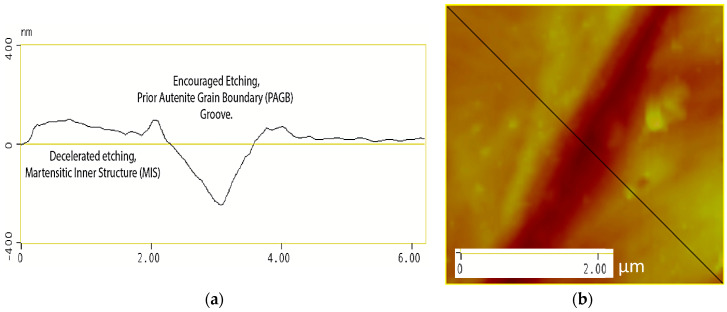
Surface analysis of the prior austenite grain boundary shown in (**a**) and the two-dimensional image shown in (**b**).

The effect of using this etching technique is shown in more detail in Figure 9a which shows the surface profile of the inner microstructure remaining close to the origin level indicating that the etching technique used does not encourage the etching of the martensitic inner structure (MIS) but accelerates the etching of the prior austenite grain boundary (PAGB), as shown in Figure 10a. 

## 4. Discussion

### 4.1. Effect of Wetting Agent 

**Figure 11:**
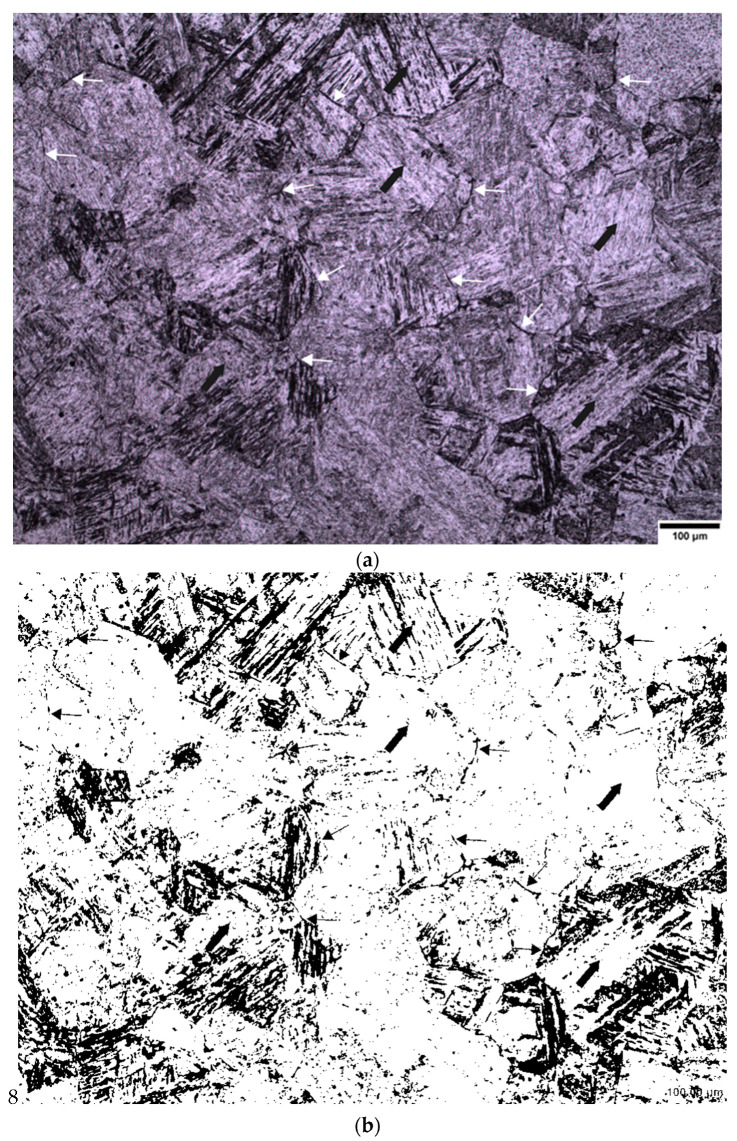
Optical micrograph showing prior austenite grain boundaries as shown by the white arrows in (**a**) and martensitic internal structure as shown by the black arrows in (**b**). Etched in saturated picric acid with sodium alkylate sulfonate (Teepol) (0.38 wt.%) carbon steel.

Wetting agents, also known as surfactants or surface-active agents, are added in small amounts to the solution to change the surface energy of a liquid or solid surface and influence interfacial interactions (modifying the etching behavior) [13]. Nelson, (1967) [14] reported that the concentration of the wetting agents gives different responses to the etching. Voort, (1995) [13] also reported the importance of wetting agents in the revealing of prior austenite grain boundaries. Many different wetting agents have been used by previous researchers to reveal the prior austenite grain boundaries and these have been shown to be effective with many different steel compositions [2,3,7,15,16] and different heat treatment conditions, e.g., as quenched, tempered or deformed. Sodium alkylate sulfonate (Teepol) in a saturated picric acid solution is the most widely used wetting agent for the delineation of prior austenite grain boundaries. Initially, sodium alkylate sulfonate was used in this study as a wetting agent to etch the prior austenite grain boundaries from an initial martensitic structure quenched from 1050 °C, which can be seen in Figure 11a,b. This wetting agent, however, proved to be unsatisfactory for the type of high purity steels which were used in this work. It can be seen from the micrograph that a few austenite grain boundaries were revealed (white arrows), but the grain size was difficult to determine as a lot of the internal martensitic structure (black arrows) was also revealed. This wetting agent was also proven to be unsuccessful in the etching of low carbon (0.08 wt.%) and high carbon (0.8 wt.%) steels as shown in Figure 2. 

**Figure 12:**
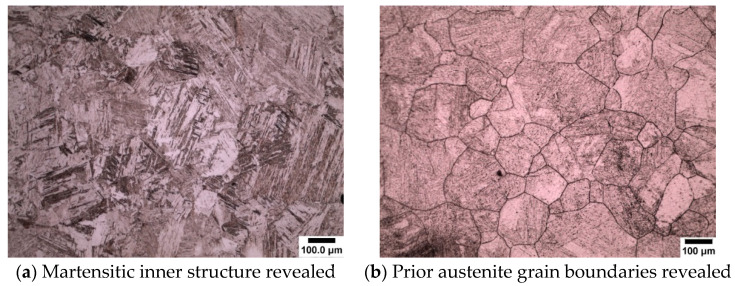
Illustrates the effect of the wetting agent on low carbon (0.08 wt.%) steel with 0.02 wt.% Nb (1150 °C). Teepol was used as the wetting agent in (**a**) and SDS used as the wetting agent in (**b**).

The new wetting agents used in this work to etch low carbon (0.08 wt.%) and medium carbon (0.38 wt.%) steels are sodium dodecyl sulfate (SDS) and sodium dodecylbenzene sulfonate (SDBS) These have been proven to give significantly good results for high carbon (0.8 wt.%) steel as shown in Figure 3, where it can be seen that the etching of the martensitic inner structure (MIS) was retarded and etching of the prior austenite grain boundaries was promoted. A side by side comparison is shown for low carbon steel quenched in iced water from 1150 °C in Figure 12, where the effect of the two wetting agents can clearly be seen. It is important to note that sodium dodecylbenzene sulfonate (SDBS) did not work for any other carbon steel concentration in this work apart from high carbon (0.8 wt.%). 

It is known that the modification of the surface tension is achieved by adding surfactants to the etchant solution [13] and increasing the temperature further reduces the surface tension between the liquid etchant and specimen surface interface [17,18]. The reduced surface tension energy by using sodium dodecyl sulfate (SDS) with the temperature between 80–90 °C provides good adhesion properties for a thick etchant layer to be formed on the steel specimen. 

### 4.2. Effect of pH Concentration 

Much of the previous research on this topic has either investigated the chemistry of the etching solution [2,3], and/or the temperature and time it took for the prior austenite grain boundaries to be successfully revealed [7]. It is also known that the time needed for effective etching to occur decreased with an increase of the temperature of the etching solution. In addition to this, the stability of the etching solution also changes with time due to the reactions occurring during etching [19]. The change in the concentration can be seen from the changes in the pH of the solution as the initial pH value prior to use was 1.22, which is acidic, but the solution becomes less acidic with use. It is known that etching of the grain boundaries is a controlled corrosive process between surface areas of different potential [18] and that an acidic solution increases the corrosion rate of the steel. Therefore, it is no surprise that when the specimen is submerged in a virgin etching solution, the etchant attacks the whole microstructure, as illustrated in Figure 6. The steel undergoes corrosion in acidic solutions which have a higher concentration of $H_{3}O^{+}$ ions. The electrons that have been generated in the anodic reaction ($Fe\to Fe^{2+}+2e^{-}$) combine with the $H_{3}O^{+}$ ions in a reduction reaction which results in the production of hydrogen gas and this in turn provides one explanation for the decrease in acidity of the etching solution with use. It is important to note that the etchant pH will also be affected by other factors such as the concentration of $Cl^{-}$ anions, derived from the HCl and potentially by atmospheric oxygen.

### 4.3. Effect of the Etchant Layer 

**Figure 13:**
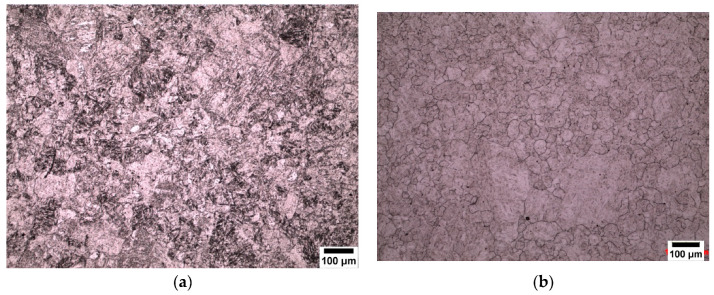
The sample shown in (**a**) has been continuously swabbed during the etching process, and the sample shown in (**b**) has been swabbed once after the formation of the etchant layer. Both samples were 0.8 wt.% carbon with 0.005 wt.% niobium and with sodium dodecyl sulfate used in the etching.

Barraclough (1973) [2] indicated that removal of the etchant layer by swabbing with cotton during the etching process resulted in a better outline of the prior austenite grain boundaries. The swabbing method used by Barraclough ensures that the etching solution reacts with a fresh surface of the specimen and a thick etchant layer does not form on the specimen. This technique has been unsuccessful in this work as shown in Figure 13a,b, where two identical specimens are shown, one which has been swabbed continuously and one which has been swabbed after 4 min when a thick etchant layer had formed on the specimen. In this work the etchant layer on the specimen was kept still in order to form an even thick etchant layer; it has been shown in Figure 4 that moving the specimen can lead to an uneven etchant layer which does not give a uniform delineation of the prior austenite grain boundaries. 

### 4.4. Novel Etching Mechanisms 

Picric acid mixed with distilled water results in a corrosive solution; therefore, when the steel specimen is immersed in a corrosive solution the grain boundaries are highly susceptible to corrode. It is known that the atoms of the metal surface are not homogenous. Some atoms are highly coordinated, as in close packed planes, whereas some are arranged at the grain boundaries and others may be foreign solute atoms such as Nb, P, Sn, Sb and As. These elements are known to segregate to the prior austenite grain boundaries [8,9] and this results in a higher energy at the grain boundaries, whereas the atoms lying within a relatively perfect close-packed structure have a lower energy [20]. Therefore, the grain boundaries are more liable to corrosive attacks than the internal microstructure.

**Figure 14:**
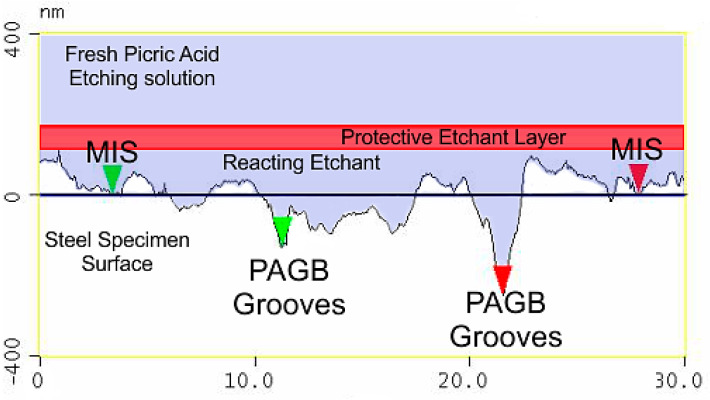
Illustration of the successful etching mechanism using atomic force microscopy (AFM).

Figure 14 illustrates the surface of the steel after the specimen has developed a thick etching layer after etching for 2 to 4 min. The prior austenite grain boundary (PAGB) grooves can be seen where selective grain boundary corrosion and retarding of the martensitic inner structure has occurred.

As explained earlier, this is due to the difference in the chemical potential of the martensitic inner structure (MIS) and the prior austenite grain boundaries (PAGB). The mechanisms of the etching process can be easily understood on the illustration. As the steel specimen is submerged into the solution the etchant starts to react with the surface of the specimen, which results in the formation of a protective etchant layer on the surface. The protective layer is a result of the corrosive reaction taking place on the surface of the specimen. During the etching process the atoms are transferred from the anodic surface to the etching solution, and this results in the formation of an insoluble metal compound which forms the etchant layer [18]. The formation of this protective etchant layer on the surface is considered to be of vital importance in the etching process as it helps stop the fresh etching solution from reacting with the specimen surface, it further accelerates the anodic process [21] and allows the reacting etchant to keep reacting with the prior austenite grains. 

**Figure 15:**
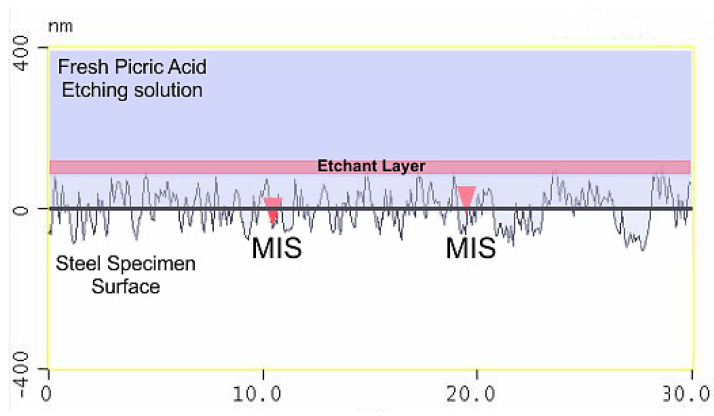
Illustration of the unsuccessful etching mechanism using atomic force microscopy (AFM).

The unsuccessful etching mechanism, in which the specimen is swabbed with cotton to remove the etchant layer, can now be analyzed in Figure 15. This results in the picric acid attacking both the martensitic inner structure and the prior austenite grain boundaries. The importance of the etching layer during the etching process and its benefits have been explained earlier. 

Other influences which are important to consider during etching are the concentration of the etchant solution, the temperature, the HCl concentration and the wetting agent used. The successful etching mechanism found in this work for high purity steel for low (0.08 wt.%) to high (0.8 wt.%) carbon steel was successful in revealing the prior austenite grain boundaries and restricting the etching of the martensitic inner structure. 

## 5. Conclusions

- The use of sodium dodecyl sulfate (SDS) as a wetting agent for high purity low (0.08 wt.%) and medium (0.38 wt.%) carbon microalloyed steel gives superior results for the delineation of prior austenite grain boundaries.
- The use of sodium dodecylbenzene sulfonate (SDBS) was shown to give good results in the revealing of prior austenite grain boundaries in high purity high carbon (0.8 wt.%) microalloyed steel.
- The etchant layer which forms on the specimen surface is an important part of the overall etching mechanism.
- Swabbing should not be done during etching after the etchant layer has formed on the surface.
- Using dummy specimens in the etchant solution prior to etching the real samples reduces the risk of needing to re-polish and re-etch.
- The roughness of the etched specimens decreases with a decrease in the acidity of the etching solution as indicated by the atomic force micrographs.

## Figures and Tables

**Table 1:** Chemical composition of 0.08 wt.% carbon steel specimens (Weight Percent).

C, wt.%	Mn, wt.%	Si, wt.%	S, wt.%	P, wt.%	Nb, wt.%
0.080	0.98	0.20	0.003	0.01	0
0.080	1.00	0.19	0.003	0.01	0.005
0.080	0.98	0.17	0.003	0.01	0.01
0.080	0.98	0.18	0.003	0.01	0.02

**Table 2:** Chemical composition of 0.38 wt.% carbon steel specimens (Weight Percent).

C, wt.%	Mn, wt.%	Si, wt.%	S, wt.%	P, wt.%	Nb, wt.%
0.38	1.00	0.20	0.003	0.018	0
0.38	0.98	0.20	0.007	0.018	0.005
0.38	1.00	0.20	0.008	0.016	0.008
0.38	1.00	0.20	0.007	0.016	0.017

**Table 3:** Chemical composition of 0.8 wt.% carbon steel specimens (Weight Percent).

C, wt.%	Mn, wt.%	Si, wt.%	S, wt.%	P, wt.%	Nb, wt.%
0.80	0.98	0.21	0.003	0.01	0.005
0.80	0.99	0.21	0.003	0.01	0
0.80	0.96	0.20	0.003	0.01	0.01
0.80	0.96	0.20	0.003	0.01	0.02
